# Study protocol: Impact of quality improvement interventions on perinatal outcomes in health facilities—a systematic review

**DOI:** 10.1186/s13643-019-1110-9

**Published:** 2019-08-15

**Authors:** Rejina Gurung, Nabila Zaka, Shyam Sundar Budhathoki, Avinash K. Sunny, Jeevan Thapa, Hong Zhou, Ashish KC

**Affiliations:** 1 Golden Community, Lalitpur, Nepal; 2 Health Section, Programme Division, UNICEF, New York, USA; 3 Department of Primary Care and Public Health, School of Public Health, Imperial College London, London, UK; 4 School of Public Health and Community Medicine, B.P. Koirala Institute of Health Sciences, Dharan, Nepal; 5 Peking University, Beijing, China; 6 Department of Women’s and Children’s Health, Uppsala University, MTC, Dag Hammarskjölds väg 14B, 1 tr, Rudbecklaboratoriet: Dag Hammaskjölds väg 20, Uppsala, Sweden

**Keywords:** Quality improvement, Health system, Health care organization, Health care workers, Perinatal care

## Abstract

**Background:**

About 5.8 million maternal deaths, neonatal deaths and stillbirths occur every year with 99% of them taking place in low- and middle-income countries. Two thirds of them could be prevented through cost-effective interventions during pregnancy, intrapartum and postpartum periods. Despite the availability of standards and guidelines for the care of mother and newborn, challenges remain in translating these standards into practice in health facilities. Although several quality improvement (QI) interventions have been systematically reviewed by the Cochrane Effective Practice and Organization of Care (EPOC) group, evidence lack on QI interventions for improving perinatal outcomes in health facilities. This systematic review will identify QI interventions implemented for maternal and neonatal care in health facilities and their impact on perinatal outcomes.

**Methods/design:**

This review will look at studies of mothers, newborn and both who received inpatient care at health facilities. QI interventions targeted at health system level (macro), at healthcare organization (meso) and at health workers practice (micro) will be reviewed. Mortality of mothers and newborn and relevant health worker practices will be assessed. The MEDLINE, Embase, World Health Organization Global Health Library, Cochrane Library and trial registries electronic databases will be searched for relevant studies from the year 2000 onwards. Data will be extracted from the identified relevant literature using Epi review software. Risk of bias will be assessed in the studies using the Cochrane risk of bias tool for randomized and observational studies. Standard data synthesis and analysis will be used for the review, and the data will be analysed using EPPI Reviewer 4.

**Discussion:**

This review will inform the global agenda for evidence-based health care by (1) providing a basis for operational guidelines for implementing clinical standards of perinatal care, (2) identify research priorities for generating evidence for QI interventions and (3) QI intervention options with lessons learnt for implementation based on the level of needed resources.

**Systematic review registration:**

PROSPERO registration number CRD42018106075

**Electronic supplementary material:**

The online version of this article (10.1186/s13643-019-1110-9) contains supplementary material, which is available to authorized users.

## Background

The call under the Sustainable Development Goals (SDGs) to reduce maternal mortality to 70 deaths per 100,000 live births, the global neonatal mortality rate to 9 per 1000 live births and global stillbirth rate to 9 per 1000 births by 2030 has led to widespread attention on increasing the coverage of live-saving interventions for mothers and babies [1–5].

If the current annual rates of reduction continue, by 2030, 116 million more mothers and babies will die with 99 million having lost development potential (including 31 million disabled), and the targets set for 2030 will be missed [6].

The availability of standard guidelines for the care of mother during pregnancy, intrapartum and postpartum period is very important to accelerate the rate of reduction in deaths [7, 8]. The translation of standard guidelines into routine health worker practices requires strong support and systems in health facilities [9–11].

A strategic pillar of the global Every Newborn Action Plan (ENAP) is to ensure the quality of care for mothers and newborn through quality improvement interventions [1, 12]. However, despite the call through the ENAP and WHO’s Ending Preventable Maternal Mortality Strategy (2015) to invest more in improving quality of care, poor health worker performance continues to compromise the care of mothers and newborn [13, 14].

Optimal health care requires much more than providing infrastructure, drugs, supplies and health care providers [15]. It needs a focus on delivering quality services that are effective, safe, people-centred, timely, equitable, integrated and efficient [16]. Quality of care is the degree to which health services increase the likelihood of desired health outcomes in line with current professional knowledge [17].

The Millennium Development Goal (MDG) period (2000–2015) saw accelerated progress on achieving global health goals [18]. Maternal mortality fell by 43% [19], although progress was unequal with preventable mortality remaining high among poor, rural and hard-to-reach populations [20]. There was significant difference in low and middle-income countries in the neonatal mortality rate for babies born in the poorest and richest households, between babies of less and most educated mothers, and between babies in rural and urban areas [21, 22]. A study conducted in India reports that although the number of institutional deliveries in Madhya Pradesh, India increased from 14% in 2005 to 80% in 2010, there was no reduction in maternal and child mortality due to poor quality care at health facilities [23].

Poor health worker performance is a fundamental cause of poor-quality care. A host of factors are associated with poor health worker performance [10, 24]. Macro-level health system factors include inadequate budgets for human resources, supplies, administrative structures and for information systems to inform decision making [25]. Meso-level health facility factors include inadequate leadership, the absence of quality improvement processes, heavy client loads, lack of health worker participation in planning and organizing work, and lack of educational opportunities for health workers [26–28]. Micro-level factors include inadequate health worker skills, motivation and job satisfaction and fear of bad clinical outcomes [29, 30]. Micro-level client-related factors include the severity of illnesses, level of patient demand and demographic factors [31]. These factors hinder the implementation of QI standards and need addressing through context-specific interventions.

There have been several systematic reviews on QI interventions for improving health worker performance [10, 11]. These have shown that the effectiveness of interventions depends on type complexity of the delivering the interventions. Different interventions have been identified for implementing care standards, with their effects varying by context, type of health worker and type of behaviour [32].

More than 100 systematic reviews conducted by Cochrane Effective Practice and Organisation of Care (EPOC) group on QI interventions for improving health worker performance [33–35]. These reviews found that the continuous education of health workers promotes good compliance with care standards, with other interventions only leading to moderate improvements in quality of care [32]. Educational outreach to health workers, educational materials and audit and feedback on performance have only had moderate effects on improving quality of care [36–38]. However, there is very little evidence on the effects of QI interventions at the macro health system level, and only limited evidence at the healthcare organization level, with a better evidence on effects at the client level [39, 40].

In 2018, to guide countries on how to better implement standards of care to achieve the health-related SDGs, the World Bank, WHO and the Organisation for Economic Co-operation and Development (OECD) identified four types of quality improvement interventions by the level of governance [16].

The availability of evidence at meso and micro levels mostly exists on tailor-made interventions, such as for diabetic care, and general health service delivery. The evidence of which QI interventions work at which level of governance for improving maternal and neonatal care is, however, limited. A 2017 systematic review on QI interventions for a small and sick newborn in low- and middle-income settings identified a number of QI interventions for improving care in sick newborn care units [40].

This systematic review will identify interventions implemented for maternal and neonatal care in health facilities and their impact on perinatal outcomes.

## Methods/design

### Reporting of review findings

This systematic review will use the reporting items for systematic reviews and meta-analyses protocols (PRISMA-P) guideline for reporting [41] and EPOC Cochrane methods [33]. The PRISMA-P checklist is provided at Additional file 1.

### Data source and search strategy

The review will search the Medline, Embase, WHO Global Health Library and Cochrane Library electronic databases from 2000 onwards to identify relevant studies. The review will also search the trial registries of WHO, WHO’s International Clinical Trials Registry Platform (ICTRP), the International Standard Randomised Controlled Trial Number (ISRCTN) and ClinicalTrials.gov for completed and ongoing studies. The searches of peer-reviewed publications will be supplemented by scanning the reference lists of relevant studies and systematic reviews. The grey literature will be searched using the databases of WHO technical reports on quality of care, UNICEF field implementation and evaluation reports and Department for International Development (DFID) technical reports. Articles with either abstracts or full text in English, Spanish, French and Chinese and published from 2000 onwards will be searched. The detailed search strategy is provided in Additional file 2.

### Eligibility criteria

The eligible population groups are systems or organizations or providers who care for mothers or newborns in inpatient settings.

### Intervention

Quality improvement is defined as the implementation of measures either together or standalone to improve the quality of care provided to mothers and/or newborn. As per the EPOC and WHO-OECD-World Bank frameworks, the QI interventions will be categorized into macro, meso and micro levels. See details in Table 1.

**Table 1:** Types of quality improvement interventions to assign for reviewing the impact of QI interventions on perinatal outcomes in health facilities

QI strategies	Description and sub-strategies
1. Targeted at health system level (macro level)	
Public reporting	Public reporting and comparative benchmarking are used to increase transparency and accountability on issues of quality and cost in health care systems by providing consumers, payers, health care organizations and providers with comparative information on performance.
Performance-based financing and contracting	Performance-based financing and contracting includes interventions that feature at least one of (i) financial incentives for providers or patients, (ii) system-wide changes in reimbursement, and (iii) changes to provider licensing or institutional accreditation requirements. There are two main types of financial incentives: • Financial incentives for health care providers include pay-for-performance, budgets that reward providers for making savings or penalize them for overspending, and incentives for practising in underserved areas or selecting vocation where there is a shortage of health professionals. • Financial incentives for recipients of health care include for specific types of behaviour (such as preventive behaviour), voucher schemes, and caps or co-payments for drugs or services that are covered by health insurance.
2. Targeted at health care organization (meso level)	
Change of organizational culture	Strategies to change organizational culture.
Continuous quality improvement and Plan-Do-Study-Act (PDSA) cycle	Continuous quality improvement aims to improve health care by (i) improving organizational processes, (ii) using structured problem-solving processes with statistical methods and measurement to diagnose problems and monitor progress, (iii) using teams of employees from multiple departments and different levels to assess quality, (iv) empowering employees to identify quality problems and improvement opportunities and to act on them, and (v) an explicit focus on clients. This covers any intervention that includes at least team or personnel changes, communications or case discussions, or total quality management and continuous improvement.
3. Targeted at health care worker practice - types of interventions (micro level)	
Continuing education meetings and workshops	Educational meetings are used for the continuing health care education of health workers to improve professional practices. Educational meetings include courses, conferences, lectures, workshops, seminars and symposia.
Facilitation	Facilitation means supporting groups of stakeholders to identify problems and responses including identifying key stakeholders who need to be engaged in order to implement changes. It involves clarifying the tasks at hand, i.e. making sense of what is going on and managing group dynamics including handling emotions in the group. Facilitators support groups to work together in a structured way and to create a climate where openness, integrity and personal values are respected.
Printed educational materials	Printed recommendations for clinical care, including clinical practice guidelines can be delivered personally or through mass mailings.
Audit and feedback	Audits and feedback include any summary of performance of health care providers or institutions. Audit and feedback processes measure professional practice or performance and then compare it to professional standards or targets to audit professional performance.
Reminders	Reminders are any patient or clinical encounter-specific information to prompt clinicians to recall information or consider a specific process of care. Manual paper reminders do not use computers in the production or delivery of reminders or in selecting target patients. They range from simple notes attached to the front of charts (‘static’ prompts) to more sophisticated reminders given under specific conditions for specific types of patients (‘dynamic’ prompts). Computer generated paper reminders are those where a computer is used either to generate paper reminders or to identify patients for whom health professionals should receive a paper reminder. Point-of-care computer reminders are those where computer reminders are delivered through a computer to health workers while they are engaged in the concerned activity.
Internet-based or computerized educational materials	The distribution of electronic recommendations for clinical care, including clinical practice guidelines. The materials are usually delivered through mass mailings and/or published on websites.
Outreach visits and mentoring	One way to improve health worker practices is to provide educational outreach visits. Trained personnel visit health workers at their points of practice and inform them how to improve their practice. The information given may include feedback about their performance, or may be based on overcoming obstacles to change. These face-to-face interactions have also been referred to as ‘university-based educational detailing’, ‘academic detailing’ and ‘educational visiting’.
Multifaceted interventions	The combinations of two or more strategies to improve practices.
Local opinion leaders	The identification and use of local opinion leaders to promote the implementation of guidelines.
Local consensus processes	Formal or informal local consensus processes to promote the implementation of guidelines.

### Comparison

The comparison group will either be no QI intervention or an intervention that has not improved the quality of care for mothers and newborn.

### Outcomes

#### Primary outcomes

- Maternal mortality refers to deaths due to complications from pregnancy or childbirth
- A stillbirth is a baby born with no signs of life at or after 28 weeks of gestation
- An antepartum stillbirth refers to where a baby dies in the womb during pregnancy and before labour or in utero 12 h before delivery
- An intrapartum stillbirth refers to where a baby dies during labour or in utero within 12 h before delivery
- First-day mortality refers to neonatal mortality within 24 h of birth
- First seven-day mortality refers to early neonatal mortality or the death of newborn babies within the first 7 days of life
- First 28-day mortality refers to late neonatal mortality or the death of newborn babies between 7 and 28 days of being born
- Pre-discharge mortality refers to where a baby dies before being discharged from health facilities

#### Secondary outcomes

- The foetal heart rate during the intrapartum period
- Infection prevention practices during labour, delivery and neonatal period
- Skin to skin contact between mother and baby in the first hour of life
- Neonatal resuscitation
- Kangaroo mother care
- Early breastfeeding
- The treatment of in-patient neonatal infection
- Duration of hospital day
- Client satisfaction

### Selection of studies

The review will consider the following types of studies on quality improvement interventions for perinatal care in health facilities [42]:

- Randomized controlled trials (RCT) at cluster and individual levels.
- Observational studies including cross-sectional studies, cohort studies and interrupted time series design studies.
- Quasi-experimental studies, including research and evaluation studies in which participants were not randomly assigned to treatment conditions, but in which comparison groups were constructed by statistical means.

### Data collection

Two of the overview authors (AK and JT) will independently screen titles and abstracts found in EPI-Review to identify reviews that may meet the inclusion criteria. Two other authors (AKC and SSB) will screen all titles and abstracts that cannot be confidently included or are excluded after the first screening to identify any additional eligible reviews. One overview author will screen the reference lists.

One overview author (AK or JT) will apply the selection criteria to the full texts of potentially eligible reviews and assess the reliability of reviews that meet all other selection criteria. Two other authors (AKC and SSB) will independently check these judgments.

### Data extraction

The review will use standard forms to extract data from research on the background of the study: interventions, participants, settings, outcomes, key findings, considerations of applicability, equity, economics, and monitoring and evaluation. The review will assess the certainty of the evidence for the main comparisons using the GRADE approach.

A standardized pre-piloted data extraction form will be developed by the team and tested on 20 articles and revised iteratively as needed. Information will be extracted on the following:

- Study setting—Region, country, location (urban/rural), level of health facility (primary, secondary or tertiary)
- Study design—Randomized control trial, observational study (cross-sectional, cohort, case-control studies), or quasi-experimental study
- Method of data analysis—Descriptive analysis of frequency, percentage, mean and standard deviation with inferential statistics through chi-squared test and logistic regression
- Quality improvement interventions—Facilitation, continuing education events, audit and feedback, reminders, checklists, outreach visits and financial incentives
- Duration of intervention
- Participants—study population, number of participants in each group and patient characteristics
- Outcomes—primary and secondary outcomes

### Quality assessment

Risk of bias and the quality of individual studies will be assessed using Cochrane risk of bias (ROB) tool for randomized studies [43] and Risk of Bias tool for non-randomized studies (RoBANS) [44] and Cochrane appraisal tool for qualitative research [45]. The use of this tool will address random sequence generation bias, allocation concealment bias, selective reporting bias, performance bias, detection bias, attrition bias and other biases. These will be further categorized as low, high or unclear risk of bias based on the type of response. This task will be completed by AKS and JT.

### Data synthesis and analysis

It is anticipated that the selected studies will have a variety of research designs that use qualitative and quantitative methods. The results from the studies will be summarized and tabulated according to the following key headings:

- Information on overarching approaches for improving the quality of health care of mothers and newborn.
- Information on whether and how the approaches for QI relate to specific signal functions for maternal and newborn care.
- The efficacy and effectiveness of the efforts to improve maternal and newborn care.
- The process and outcomes used as measures of quality improvement.
- Where possible, results will be disaggregated according to geography (regional, sub-national levels), wealth quintile, rural or urban setting, public or private health facility and type of health facility.

### Meta-biases

Research studies can have implementation and design bias, which will be referred to as meta-biases [46, 47]. Funnel plots will be done when a number of studies go beyond 10 to assess reporting bias [48].

### Confidence in cumulative evidence

GRADE criteria will be used to assess the quality of studies and reporting done in the studies [49]. GRADE provides a clear and comprehensive methodology for rating and summarizing quality of evidence and thus supports management recommendations [50]. GRADE assigns high, moderate, low, and very low levels of evidence quality. For qualitative studies, the GRADE-CERQual (Grading of Recommendations Assessment, Development and Evaluation-Confidence in Evidence from Reviews of Qualitative research) will be used [51].

Randomized trials generally begin as high-quality evidence and observational studies as low-quality evidence. The quality of evidence may be subsequently downgraded as a result of limitations in study design or implementation, imprecision of estimates (wide confidence intervals), variability in results, indirectness of evidence or publication bias. Quality may be upgraded because of a very large magnitude of effect, a dose-response gradient and if all plausible biases would reduce an apparent treatment effect. Quality assessments will be undertaken by two independent researchers. Any discrepancies will be resolved through discussion with a third reviewer.

The framework will thus examine the quality of methodology, the relevance of methodology and the relevance of evidence to the issue under review. An average of these weightings will be taken to establish the overall weight of evidence of each study.

## Discussion

This systematic review has three implications. First, it will identify evidence on QI interventions implemented to improve the care of mothers and newborn in health facilities and provide further evidence on the impact of QI interventions on quality of care. The evidence on the impact on care on maternal and neonatal care will enable an implementation framework on quality improvement to be developed. This framework can then be used to develop overall operational guidelines for implementing standards of care for mothers and newborn in different health care settings.

Second, the types of QI interventions identified will indicate gaps in the evidence base for informing decision-making on QI interventions. The review will identify research gaps on QI interventions for improving care to identify subjects that need researching.

Third, the evidence of the impact of QI interventions on improving care will provide guidance to governments and other stakeholders on the resources needed to implement QI interventions for the more effective care of mothers and babies.

The QI interventions in this review will discuss the context of implementing the interventions. The Consolidated Framework for Implementation Research (CFIR) [52, 53] with its five domains; namely, characteristics of the intervention, individuals, inner setting, outer setting and implementation process will help facilitate the discussion of the context in which the intervention has been implemented. Many governments struggle to justify the investments needed to further improve maternal and neonatal health care against competing demands to spend more on combatting non-communicable diseases, re-emerging infectious diseases and other priorities. There is a need for an implementation framework for QI interventions for maternal and neonatal care. This systematic review will be an evidence-based reference document for WHO, UNICEF and other global health care partners to develop implementation guideline.

## Additional files

Additional file 1: PRISMA-P Checklist: Impact of Quality improvement intervention(s) on perinatal outcome in health facilities: a systematic review. (DOCX 20 kb)

Additional file 2: Search strategy. (DOCX 13 kb)

## Abbreviations

EPOC: Effective Practice and Organization of Care
GRADE: Grading of Recommendations Assessment, Development and Evaluation
MDG: Millennium Development Goal
PRISMA: Preferred Reporting Items for Systematic Reviews and Meta-Analyses
QI: Quality improvement
SDG: Sustainable Development Goal
WHO: World Health Organization
