# Supplementary material for: Study protocol: Impact of quality improvement interventions on perinatal outcomes in health facilities—a systematic review
Source: Syst Rev. 2019 Aug 15;8:205. doi: 10.1186/s13643-019-1110-9 (PMC6694558; doi:10.1186/s13643-019-1110-9)
Supplement: Supplementary file 2 — Search strategy. (DOCX 13 kb) [file 13643_2019_1110_MOESM2_ESM.docx]

Date 19.8.2018

(((((((((((((((("Quality Improvement"[Mesh:noexp] OR "Congresses as Topic"[Mesh:noexp]) OR "Organizational Innovation"[Mesh:noexp]) OR "Education"[Mesh:noexp]) OR "Mentoring"[Mesh]) OR "Education, Medical, Continuing"[Mesh]) OR "Inservice Training"[Mesh]) OR "Checklist"[Mesh]) OR "Reimbursement, Incentive"[Mesh]) OR "Quality Indicators, Health Care"[Mesh:noexp]) OR "Quality of Health Care"[Mesh:noexp]) OR "Benchmarking"[Mesh]) OR "Clinical Audit"[Mesh]) OR "Feedback"[Mesh]) OR "Reminder Systems"[Mesh]) OR "Social Facilitation"[Mesh]) OR ("Public reporting"[All Fields] OR "benchmarking"[MeSH Terms] OR "benchmarking"[All Fields] OR ("contracts"[MeSH Terms] OR "contracts"[All Fields] OR "contracting"[All Fields]) OR ("change"[All Fields] AND ("organizational culture"[MeSH Terms] OR ("organizational"[All Fields] AND "culture"[All Fields]) OR "organizational culture"[All Fields])) OR (Continuous[All Fields] AND ("quality improvement"[MeSH Terms] OR ("quality"[All Fields] AND "improvement"[All Fields]) OR "quality improvement"[All Fields]) OR PDSA[All Fields]) OR (("education, continuing"[MeSH Terms] OR ("education"[All Fields] AND "continuing"[All Fields]) OR "continuing education"[All Fields] OR ("continuing"[All Fields] AND "education"[All Fields])) AND meetings[All Fields] AND ("education"[MeSH Terms] OR "education"[All Fields] OR "workshops"[All Fields])) OR (facilitation[tiab] OR "social facilitation"[MeSH Terms]) OR "Printed educational materials"[All Fields] OR "audit and feedback"[All Fields] OR (reminder[All Fields] OR reminders[All Fields] OR "Reminder Systems"[Mesh]) OR (Internet-based[All Fields] OR computerized[All Fields] AND (educational[All Fields] AND "materials"[All Fields])) OR ("mentoring"[MeSH Terms] OR "mentoring"[All Fields]) OR (outreach[All Fields] AND educational[All Fields] AND visits[All Fields]) OR (Multifaceted[All Fields] AND interventions[All Fields]) OR "Local opinion leader"[All Fields] OR "local consensus"[All Fields])) AND ((((("Mothers"[Mesh:noexp] OR "Infant, Newborn"[Mesh:noexp]) OR "Perinatal Care"[Mesh]) OR "Maternal Health Services"[Mesh:noexp]) OR "Intensive Care, Neonatal"[Mesh]) OR ((("health services"[MeSH Terms] OR ("health"[All Fields] AND "services"[All Fields]) OR "health services"[All Fields]) OR ("health facilities"[MeSH Terms] OR ("health"[All Fields] AND "facilities"[All Fields]) OR "health facilities"[All Fields] OR ("health"[All Fields] AND "facility"[All Fields]) OR "health facility"[All Fields])) AND (perinatal[All Fields] OR ("infant, newborn"[MeSH Terms] OR ("infant"[All Fields] AND "newborn"[All Fields]) OR "newborn infant"[All Fields] OR "neonatal"[All Fields]) OR ("mothers"[MeSH Terms] OR "mothers"[All Fields] OR "maternal"[All Fields]))))

[11144](https://www.ncbi.nlm.nih.gov/pubmed?cmd=HistorySearch&querykey=1)

Filters activated:

**Randomized Controlled Trial,**

**Observational Study,**

**Comparative Study,**

**Validation Studies,**

**Evaluation Studies,**

**Multicenter Study,**

**Clinical Study**,

Publication date from 2000/01/01, Humans. [Clear all](https://www.ncbi.nlm.nih.gov/pubmed) to show 11144 items.

(((((((((((((((("Quality Improvement"[Mesh:noexp] OR "Congresses as Topic"[Mesh:noexp]) OR "Organizational Innovation"[Mesh:noexp]) OR "Education"[Mesh:noexp]) OR "Mentoring"[Mesh]) OR "Education, Medical, Continuing"[Mesh]) OR "Inservice Training"[Mesh]) OR "Checklist"[Mesh]) OR "Reimbursement, Incentive"[Mesh]) OR "Quality Indicators, Health Care"[Mesh:noexp]) OR "Quality of Health Care"[Mesh:noexp]) OR "Benchmarking"[Mesh]) OR "Clinical Audit"[Mesh]) OR "Feedback"[Mesh]) OR "Reminder Systems"[Mesh]) OR "Social Facilitation"[Mesh]) OR ("Public reporting"[All Fields] OR "benchmarking"[MeSH Terms] OR "benchmarking"[All Fields] OR ("contracts"[MeSH Terms] OR "contracts"[All Fields] OR "contracting"[All Fields]) OR ("change"[All Fields] AND ("organizational culture"[MeSH Terms] OR ("organizational"[All Fields] AND "culture"[All Fields]) OR "organizational culture"[All Fields])) OR (Continuous[All Fields] AND ("quality improvement"[MeSH Terms] OR ("quality"[All Fields] AND "improvement"[All Fields]) OR "quality improvement"[All Fields]) OR PDSA[All Fields]) OR (("education, continuing"[MeSH Terms] OR ("education"[All Fields] AND "continuing"[All Fields]) OR "continuing education"[All Fields] OR ("continuing"[All Fields] AND "education"[All Fields])) AND meetings[All Fields] AND ("education"[MeSH Terms] OR "education"[All Fields] OR "workshops"[All Fields])) OR (facilitation[tiab] OR "social facilitation"[MeSH Terms]) OR "Printed educational materials"[All Fields] OR "audit and feedback"[All Fields] OR (reminder[All Fields] OR reminders[All Fields] OR "Reminder Systems"[Mesh]) OR (Internet-based[All Fields] OR computerized[All Fields] AND (educational[All Fields] AND "materials"[All Fields])) OR ("mentoring"[MeSH Terms] OR "mentoring"[All Fields]) OR (outreach[All Fields] AND educational[All Fields] AND visits[All Fields]) OR (Multifaceted[All Fields] AND interventions[All Fields]) OR "Local opinion leader"[All Fields] OR "local consensus"[All Fields])) AND ((((("Mothers"[Mesh:noexp] OR "Infant, Newborn"[Mesh:noexp]) OR "Perinatal Care"[Mesh]) OR "Maternal Health Services"[Mesh:noexp]) OR "Intensive Care, Neonatal"[Mesh]) OR ((("health services"[MeSH Terms] OR ("health"[All Fields] AND "services"[All Fields]) OR "health services"[All Fields]) OR ("health facilities"[MeSH Terms] OR ("health"[All Fields] AND "facilities"[All Fields]) OR "health facilities"[All Fields] OR ("health"[All Fields] AND "facility"[All Fields]) OR "health facility"[All Fields])) AND (perinatal[All Fields] OR ("infant, newborn"[MeSH Terms] OR ("infant"[All Fields] AND "newborn"[All Fields]) OR "newborn infant"[All Fields] OR "neonatal"[All Fields]) OR ("mothers"[MeSH Terms] OR "mothers"[All Fields] OR "maternal"[All Fields])))) AND ((Randomized Controlled Trial[ptyp] OR Observational Study[ptyp] OR Comparative Study[ptyp] OR Validation Studies[ptyp] OR Evaluation Studies[ptyp] OR Multicenter Study[ptyp] OR Clinical Study[ptyp]) AND ("2000/01/01"[PDAT] : "3000/12/31"[PDAT]) AND "humans"[MeSH Terms])

**1612**
